# Abscess of Temporal Region from Gunshot Injury

**Published:** 1872-01

**Authors:** J. W. Southworth

**Affiliations:** Toledo, O.


ART. VI.—*Abcess of Temporal Region from Gunshot Injury:—Extraction of ball after Four Years Lodgment Behind Zygomatic Arch. By* J. W. SOUTHWORTH, M. D. *Toledo, O.*

Mr. G. W. E.—mulatto—aged 28, of stout build, was struck by a partially spent ball or balls (?) in the temple, while serving in the late war,—on the 7th of June, 1864. Says the hospital surgeon told him a ball was found and taken out. "Wound did not heal until after a piece of bone came out." Cicatrix was always tender, and he was troubled with some headache. In the summer of 1865 had increase of pain and throbbing, with vertigo after stooping, eventuating in an abcess which was opened and treated in the usual manner by his attendant. It healed soon. Cicatrix still tender. Headache and vertigo not very troublesome, until the summer of 1866, when another abcess formed in same situation; was treated as usual by another practitioner. The abcess again healing entirely—(cicatrix was still tender] and remained so until June, 1868. The patient having in the meantime more or less throbbing headache during the hot season when working in the sun. Frequent applications of cold water releived it and the vertigo as well.

On June 8th, 1868, he applied to me (at Adrian, Mich.) giving previous history as stated, All the premonitory symptoms of an abcess being present, viz: tenderness, throbbing pain and slight swelling, and fever. He was treated with a brisk cathartic of calomel, podophylin and nitre, with a mixture of veratrum, antimony and dovers powder to control febrile action, and bowels kept open with a saline draught at night. All the symptoms increased in violence—vision impaired on affected side, severe pain on moving lower jaw; intense throbbing pain in temple and swelling increased, with œdema. Made an incision at the most prominent point on the 10th, but no pus followed. Constitutional and local symptoms abated on the evening of the 11th, and on the 12th it was again incised, when a free flow of pus followed, with great relief to pain and tension.

Having surmised the existance of some foreign substance, either a necrosed spicula of bone or a fragment of the ball said to have been extracted, I explored the cavity with Nelaton's porcelain tipped probe. It passed down about 2 1-2 inches and struck a solid substance behind the zygomatic arch which discolored the porcelain, proving it to be lead. The incision was immediately enlarged sufficiently to admit the finger,—cutting across the anterior branch of the temporal artery [which was ligatured] and dividing the anterior fibres of the temporal muscle, *behind which* the forceps were passed down to the ball, which was fortunately seized at the best advantage and extracted on the first trial. It weighed 3-4 of an ounce, was indented, or furrowed rather, on one side, and a little rough on the other. The abcess healed rapidly, patient remains well, the obscuration of vision, and the headache, &c., having gradually ceased. Patient objected to taking chloroform or it would have been given.

